# Mortality risk stratification in contemporary myocardial infarction–related cardiogenic shock: development of AMICS-ADMIT prediction model and benchmarking of established scores

**DOI:** 10.1016/j.ijcha.2026.102029

**Published:** 2026-10-14

**Authors:** Klevis Mihali, Birgit Markus, Kornelia Neuner, Georgios Chatzis, Lukas Harbaum, Mykyta Fomin, Maryana Choukeir, Styliani Syntila, Bernhard Schieffer, Julian Kreutz

**Affiliations:** Philipps-Universität Marburg, Department of Cardiology, Angiology, and Intensive Care Medicine, Baldingerstraße, Marburg, 35043, Germany

**Keywords:** Cardiogenic shock, Acute myocardial infarction, Risk stratification, Mortality prediction, Mechanical circulatory support

## Abstract

**Background:**

Mortality risk stratification in acute myocardial infarction complicated by cardiogenic shock (AMI-CS) remains challenging with heterogeneous temporary mechanical circulatory support (tMCS) use. We developed and temporally validated the admission-based AMICS-ADMIT model and bedside score and benchmarked them against established scores.

**Methods:**

This retrospective single-centre study, reported according to TRIPOD+AI, included 479 consecutive patients with AMI-CS undergoing percutaneous coronary intervention (PCI) between January 2017 and July 2025. The primary endpoint was in-hospital mortality. AMICS-ADMIT was developed using multivariable logistic regression in the 2017–2022 cohort, internally evaluated by bootstrap resampling, and temporally validated in the 2023–2025 cohort. Missing data were multiply imputed. Performance was assessed using discrimination, calibration, and Brier scores. Benchmarking against CardShock, IABP-SHOCK II, and GRACE was restricted to temporal validation; tMCS-stratified performance was exploratory.

**Results:**

In-hospital mortality was 42.6% (204/479). The final model included age, left ventricular ejection fraction, prior cardiopulmonary resuscitation, estimated glomerular filtration rate, vasoactive–inotropic score, and ST-segment elevation status. The continuous model achieved AUCs of 0.809 (95% CI 0.764–0.855) in development and 0.834 (95% CI 0.769–0.899) in temporal validation, with Brier scores of 0.176 and 0.167 and favourable calibration. The bedside score achieved AUCs of 0.777 (95% CI 0.728–0.826) and 0.775 (95% CI 0.699–0.851), respectively, with stepwise mortality separation across predefined risk categories. Discrimination did not differ significantly from CardShock, IABP-SHOCK II, or GRACE.

**Conclusions:**

AMICS-ADMIT showed stable temporal performance and clinically interpretable early risk stratification in contemporary PCI-treated AMI-CS. External multicentre validation is required before clinical implementation.

## Graphical abstract

**Figure:**
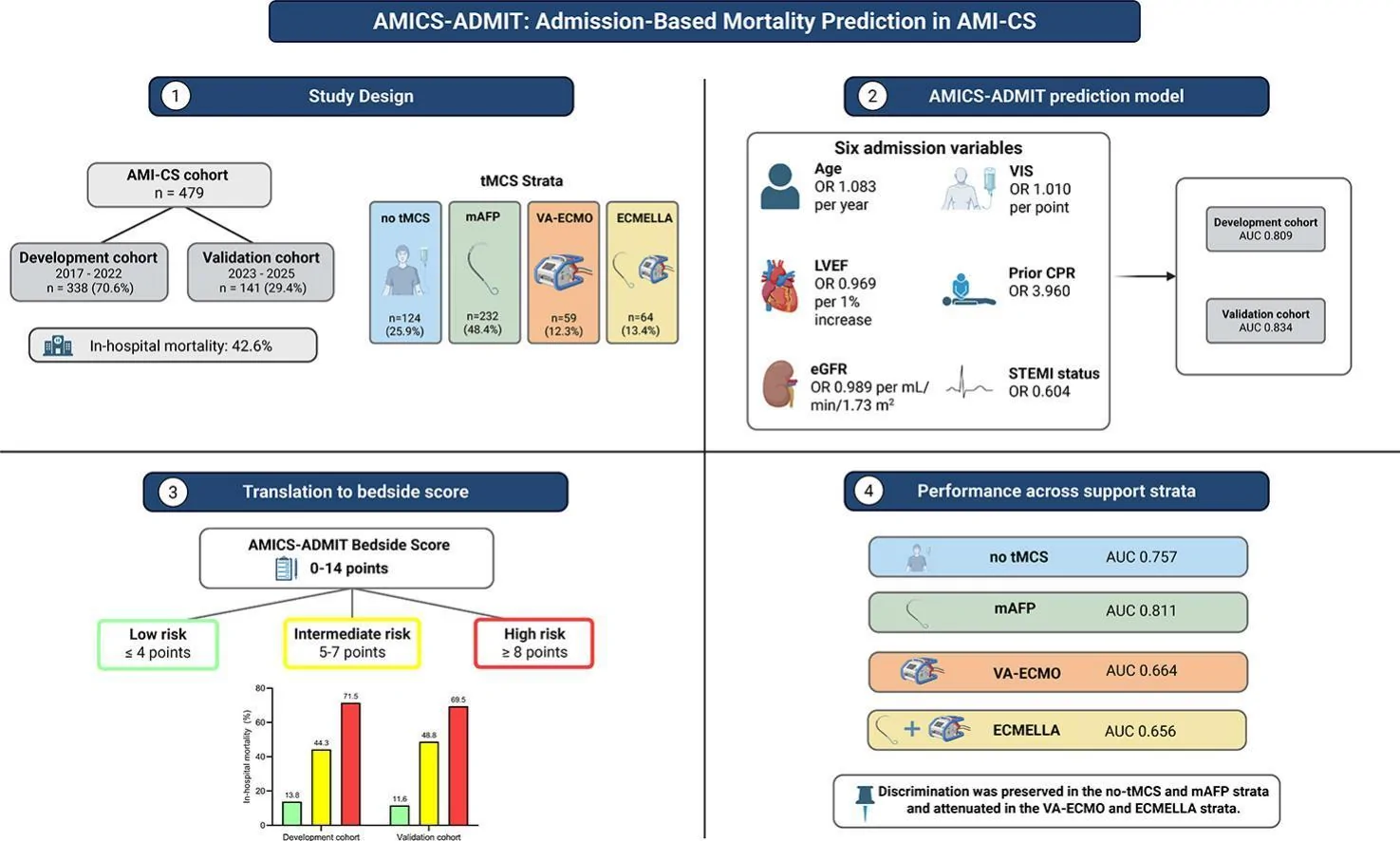

## 1 Introduction

Acute myocardial infarction complicated by cardiogenic shock (AMI-CS) remains one of the most lethal syndromes in contemporary cardiovascular medicine, with short-term mortality still approaching 40–50% despite emergency networks, early coronary angiography, revascularization, and advances in cardiovascular intensive care [1], [2], [3]. Current guidelines therefore emphasize rapid recognition of shock, immediate treatment of the underlying ischaemic trigger, correction of hypoperfusion, and structured escalation of care [4], [5]. However, decisions regarding shock-team activation, escalation of care, and circulatory support are often made within the first minutes to hours after admission, before the trajectory of multiorgan failure and treatment response becomes apparent. The therapeutic landscape of AMI-CS has changed substantially with increasing use of temporary mechanical circulatory support (tMCS), including microaxial flow pumps (mAFP), veno-arterial extracorporeal membrane oxygenation (VA-ECMO), and combined unloading approaches such as ECMELLA [6]. These strategies have expanded haemodynamic treatment options but also increased clinical heterogeneity. Recent randomized evidence underscores that tMCS is not a uniform therapeutic concept: in selected patients with ST-segment elevation myocardial infarction (STEMI)-related CS, routine mAFP support reduced mortality but increased complications, whereas early unselected VA-ECMO did not reduce 30-day mortality and increased bleeding and vascular events [7], [8].

Several risk scores have been developed for CS or acute coronary syndromes (ACS), including CardShock, IABP-SHOCK II, GRACE, and ENCOURAGE [9], [10], [11], [12]. However, these tools were derived in different clinical contexts, treatment eras, and patient populations, and their transportability to contemporary AMI-CS across the full spectrum of current support strategies remains uncertain. This creates a need for an early admission-based model that estimates mortality risk using variables available during initial hospital care, before the subsequent clinical trajectory is fully established. Ideally, this model should rely on routinely available variables, provide reliable early risk estimation, remain stable across evolving treatment eras, and be translatable into a simple bedside score that supports baseline risk assessment, clinical communication, cohort characterization, and future risk-guided studies without replacing dynamic reassessment or device-specific decision-making.

We aimed to develop and temporally validate the AMICS-ADMIT model for early in-hospital mortality risk stratification in a contemporary PCI-treated AMI-CS cohort in the tMCS era. To facilitate bedside application, the admission-based model was translated into a simplified bedside score using variables obtained during early hospital care. We further descriptively benchmarked AMICS-ADMIT against established risk scores and explored its performance across no-tMCS, mAFP, isolated VA-ECMO, and ECMELLA strata.

## 2 Materials and methods

### 2.1 Study design, population, and data collection

This retrospective, single-centre observational cohort study was conducted at the Department of Cardiology, Angiology and Intensive Care Medicine, University Hospital of Marburg, Germany. The study was approved by the Ethics Committee of the Faculty of Medicine, Philipps University Marburg (No. 26-284 RS; 14 August 2026) and was conducted in accordance with the Declaration of Helsinki. Owing to the retrospective, non-interventional design, the requirement for individual informed consent was waived. Consecutive adult patients (≥18 years) admitted between January 2017 and July 2025 with AMI-CS were screened for inclusion. AMI included STEMI and non-ST-segment elevation myocardial infarction (NSTEMI) [13]. CS was defined according to contemporary European Society of Cardiology criteria, including persistent hypotension accompanied by signs of end-organ hypoperfusion attributable to primary cardiac dysfunction [14]. Only patients retrospectively assessed as fulfilling Society for Cardiovascular Angiography and Interventions (SCAI) shock stage C or higher based on the available clinical documentation were included, thereby restricting the study population to patients with manifest or advanced CS [15]. Patients who did not undergo coronary angiography and those who underwent coronary angiography without percutaneous coronary intervention (PCI) were excluded. All patients were managed within a standardized institutional shock algorithm including urgent coronary revascularization when indicated and stepwise escalation to tMCS at the discretion of the treating physicians. Mechanical support strategies included isolated mAFP support (Impella®, Abiomed, Johnson & Johnson MedTech, Danvers, MA, USA), VA-ECMO (Cardiohelp®, Getinge, Gothenburg, Sweden), or combined support (ECMELLA). Patients were categorized according to the tMCS strategy used during the index hospitalization as no tMCS, isolated mAFP, isolated VA-ECMO, or ECMELLA. ECMELLA was defined as simultaneous support with both mAFP and VA-ECMO. Patients were assigned to the ECMELLA group only when both devices were in use concurrently; sequential non-overlapping use of mAFP and VA-ECMO did not meet the definition of ECMELLA.

The primary endpoint was all-cause in-hospital mortality. In-hospital mortality was determined from the electronic medical record and hospital discharge documentation and was defined as death from any cause occurring during the index hospitalization. Because mortality represents an objective endpoint and data collection was retrospective, no formal blinding of outcome assessment to predictor information was performed. Clinical, laboratory, echocardiographic, and procedural data were extracted from electronic medical records. Age was recorded at hospital admission. Prior cardiopulmonary resuscitation (CPR) was defined as any CPR performed before or at hospital admission. STEMI status was determined from the admission electrocardiogram according to contemporary guideline criteria. Left ventricular ejection fraction (LVEF) was obtained from the earliest clinically available echocardiographic examination after hospital admission. Estimated glomerular filtration rate (eGFR) was calculated from the first creatinine measurement obtained from the blood sample collected at intensive care unit (ICU) admission using the 2021 race-free CKD-EPI creatinine equation [16]. The vasoactive–inotropic score (VIS) was calculated from vasoactive and inotropic medication doses documented at ICU admission using the conventional formula: dopamine dose (μg/kg/min) + dobutamine dose (μg/kg/min) + 100 × epinephrine dose (μg/kg/min) + 10 × milrinone dose (μg/kg/min) + 100 × norepinephrine dose (μg/kg/min) + 10,000 × vasopressin dose (units/kg/min). At the index assessment, no patients received dopamine, milrinone, or vasopressin; these components therefore contributed zero to the VIS in all patients. For the purposes of this study, ‘admission-based’ refers to variables obtained during the initial hospital and ICU assessment and should not be interpreted as indicating that all predictors were measured before CPR, PCI, vasoactive treatment, or tMCS initiation. Because predictor data were retrospectively extracted from clinical records, predictor assessment was not formally blinded to the outcome. The Charlson Comorbidity Index (CCI) was calculated from documented pre-existing comorbidities according to the original weighted scoring system, with higher values indicating a greater burden of chronic disease [17]. This study was reported in accordance with the TRIPOD+AI statement for clinical prediction model studies [18].

### 2.2 Model development and derivation of the AMICS-ADMIT bedside score

Candidate predictors were restricted to routinely available variables documented during the early hospital assessment and comprised age, sex, known coronary artery disease, chronic heart failure, eGFR, LVEF, STEMI status, prior CPR, CPR duration, initial PEA/asystole, lactate, mean arterial pressure, and VIS. These variables were initially screened for association with in-hospital mortality, using p < 0.05 as the threshold for eligibility for multivariable modelling. The corresponding unadjusted associations are provided in Supplementary Table 1. Initial screening and subsequent model selection were performed exclusively in the 2017–2022 development cohort; the 2023–2025 temporal validation cohort was not used for predictor selection or coefficient estimation. Variables meeting this criterion and considered clinically appropriate for early risk assessment were entered into a multivariable logistic regression model. Backward elimination was subsequently performed, with variables showing p ≥ 0.05 sequentially removed unless their retention was justified on clinical grounds. eGFR and STEMI status were retained despite borderline statistical significance in the original model because they represented clinically relevant and complementary pathophysiological domains of AMI-CS. This initial selection procedure resulted in the six-predictor set comprising age, LVEF, eGFR, VIS, prior CPR, and STEMI status. Following revision of the missing-data strategy, this six-predictor set was fixed before multiple-imputation model estimation. Model development was performed in the entire 2017–2022 development cohort (n = 338; 143 in-hospital deaths) after multiple imputation of missing predictor data. Fifty imputed datasets were generated under the missing-at-random assumption using fully conditional specification, with predictive mean matching based on five nearest neighbours for continuous variables and 20 iterations per dataset. The imputation model included age, LVEF, eGFR, VIS, prior CPR, STEMI status, in-hospital mortality, death within 24 hours, tMCS strategy, lactate, base excess, haemoglobin, and the CCI. Age, LVEF, eGFR, VIS, prior CPR, and STEMI status were entered simultaneously into the regression model in each imputed development dataset, with no further variable selection performed. Logistic regression coefficients and standard errors were pooled across the imputed datasets using Rubin’s rules.

The assumption of linearity in the log-odds was assessed for age, LVEF, eGFR, and VIS in the multiply imputed development cohort using Box–Tidwell transformation terms, with no evidence of relevant nonlinearity for any of the four continuous predictors. The study size was determined by the number of consecutive eligible patients treated during the predefined study period; no formal a priori sample-size calculation was performed. In a post hoc assessment according to the framework proposed by Riley et al., based on 13 candidate predictor parameters, an outcome prevalence of 42.3%, and an apparent C-statistic of 0.809, the estimated minimum sample size was 376 patients with approximately 160 events [19]. No coefficient shrinkage was applied. As an additional internal validation analysis, bootstrap resampling was performed for the fixed six-predictor model across the 50 multiply imputed development datasets. Two hundred bootstrap samples were generated within each imputed dataset, resulting in 10,000 bootstrap model fits. In each bootstrap sample, the six-predictor logistic regression model was refitted and evaluated both in the bootstrap sample and in the corresponding original imputed dataset. Mean optimism was estimated for discrimination, Brier score, and calibration slope, and was used to derive optimism-corrected performance estimates. Bootstrap distributions of the regression coefficients were additionally examined to assess predictor stability. Regression coefficients from the final model were used to estimate predicted in-hospital mortality probabilities, defining the continuous AMICS-ADMIT prediction model. For bedside application, the final model was converted into the simplified additive AMICS-ADMIT bedside score using the six retained admission predictors. Continuous variables were categorized using clinically interpretable thresholds reflecting age decades, left ventricular systolic dysfunction, renal impairment, and VIS intensity. The bedside score was constructed as a coefficient-informed clinical simplification rather than by dividing all pooled regression coefficients by a single constant; therefore, no universal scaling factor or uniform rounding rule was applied. Integer point allocation was guided by the direction and relative magnitude of the pooled regression coefficients together with clinical interpretability. For the continuous predictors, clinically meaningful thresholds were used to define ordered categories, and one point was assigned for each worsening category relative to the lowest-risk reference category. Consequently, age and LVEF, each comprising four categories, contributed 0–3 points, whereas eGFR and VIS, each comprising three categories, contributed 0–2 points. Among the binary predictors, prior CPR showed the strongest adverse association with mortality and was assigned 3 points, whereas STEMI showed a smaller inverse association; accordingly, STEMI received 0 points and its absence received 1 point. This coefficient-informed simplification produced a total score ranging from 0 to 14 points, with higher values indicating greater predicted in-hospital mortality risk.

### 2.3 Model performance, validation, and comparative analyses

Discrimination of the continuous AMICS-ADMIT model and bedside score was assessed using the area under the receiver operating characteristic curve (AUC) with 95% confidence intervals (CIs). Overall predictive accuracy was quantified using the Brier score. Calibration of the continuous model was evaluated using the calibration slope, calibration-in-the-large, the jointly estimated calibration intercept, and visual calibration plots. Temporal validation was performed by applying the pooled coefficients and intercept derived in the 2017–2022 development cohort to the 2023–2025 cohort without coefficient re-estimation or recalibration. Missing predictor values in the temporal validation cohort were handled using 50 separately generated imputed datasets. To preserve temporal independence and approximate prospective application, in-hospital mortality and death within 24 hours were not included in the validation-cohort imputation model. The imputation model included age, LVEF, eGFR, VIS, prior CPR, STEMI status, lactate, base excess, haemoglobin, and the CCI. The fixed development-model coefficients were applied to each imputed validation dataset, and patient-level predicted probabilities were averaged across the 50 datasets. The linear predictor of the primary six-predictor model was calculated as LP = −4.93202 + 0.07946 × age − 0.03127 × LVEF − 0.01153 × eGFR + 0.01041 × VIS + 1.37630 × prior CPR − 0.50489 × STEMI. Prior CPR and STEMI were coded as 1 for yes and 0 for no. The predicted probability was calculated as p = 1/[1 + exp(−LP)] (Supplementary Table 2). The development and temporal validation cohorts were derived from the same centre and were defined using identical eligibility criteria, predictor definitions, data sources, and outcome assessment. They differed only with respect to the calendar period of treatment: 2017–2022 for model development and 2023–2025 for temporal validation. To examine the influence of LVEF-related missingness, an otherwise identical five-predictor model excluding LVEF was re-derived in the multiply imputed development cohort. Its pooled coefficients were subsequently applied, without re-estimation or recalibration, to the separately imputed temporal validation cohort. Performance of the primary model was summarized using the AUC, Brier score, calibration slope, calibration-in-the-large, and jointly estimated calibration intercept. The no-LVEF sensitivity model was assessed using the AUC and Brier score. As an additional sensitivity analysis, the same six-predictor logistic regression model was refitted in development-cohort patients with complete data for all six predictors, without further variable selection; model discrimination and overall predictive accuracy were assessed using the AUC and Brier score.

The predictor categories, integer point weights, and risk-group cut-offs were defined using the 2017–2022 development cohort and subsequently applied unchanged to the 2023–2025 temporal validation cohort. Patients were categorized as low risk (≤4 points), intermediate risk (5–7 points), or high risk (≥8 points). These cut-offs were selected pragmatically in the development cohort to provide clinically interpretable strata with adequate group sizes and were not used in fitting the continuous model. The total bedside score was calculated separately within each of the 50 imputed development datasets, and its relationship with in-hospital mortality was estimated using logistic regression. The intercept and score coefficient were pooled using Rubin’s rules, yielding the fixed score-to-risk equation logit(p) = −3.16665 + 0.47386 × AMICS-ADMIT score. This equation was applied without coefficient re-estimation or recalibration in each of the 50 separately imputed temporal validation datasets, and patient-level predicted probabilities were averaged across imputations. Bedside-score performance was assessed in the full multiply imputed development and temporal validation cohorts using the AUC, Brier score, calibration slope, calibration-in-the-large, and jointly estimated calibration intercept. The predefined risk groups were compared using logistic regression within each imputed dataset, with the low-risk group serving as the reference category; estimates were pooled using Rubin’s rules.

Observed in-hospital mortality was summarized as proportions across AMICS-ADMIT risk categories and tMCS strategies. Principal benchmarking of the AMICS-ADMIT bedside score against CardShock, IABP-SHOCK II, and GRACE was restricted to the 2023–2025 temporal validation cohort using identical paired samples, and paired AUCs were compared using DeLong’s test. Separately, exploratory analyses across tMCS strata were performed in the overall cohort. For AMICS-ADMIT, patient-level predicted probabilities were averaged across the 50 multiply imputed datasets, whereas established scores were evaluated using available cases. ENCOURAGE was evaluated only in patients receiving isolated VA-ECMO. Given differences in derivation populations, prediction horizons, and intended use, these treatment-stratified analyses were considered descriptive and were not interpreted as formal head-to-head comparisons or evidence of score superiority.

### 2.4 Statistical analysis

Continuous variables were summarized as mean ± standard deviation or median [interquartile range], depending on their distribution, and categorical variables as n (%). Between-group comparisons were performed using Student’s t-test or the Mann–Whitney U test for continuous variables and Pearson’s chi-square test for categorical variables. Logistic regression modelling, primary receiver operating characteristic analyses, calibration analyses, and multiple imputation were performed in SPSS. Comparative receiver operating characteristic analyses and paired AUC comparisons using DeLong’s test were conducted in R. No class-imbalance correction, such as outcome weighting, oversampling, or undersampling, was applied, and no formal analyses of model fairness were performed. All statistical tests were two-sided, and p < 0.05 was considered statistically significant. Statistical analyses were performed using IBM SPSS Statistics version 29.0.2.0 (IBM Corp., Armonk, NY, USA) and R version 4.5.2 (R Foundation for Statistical Computing, Vienna, Austria). Figures were generated using GraphPad Prism version 11.0 (GraphPad Software, Boston, MA, USA).

## 3 Results

### 3.1 Patient characteristics and in-hospital outcomes

A total of 563 patients with AMI-CS fulfilling SCAI shock stage C or higher were identified during the study period. Of these, 36 patients who did not undergo coronary angiography and 48 patients who underwent coronary angiography without PCI were excluded. The final study population therefore comprised 479 patients treated between January 2017 and July 2025 (Fig. 1). According to treatment strategy, 124 patients (25.9%) were managed without tMCS, 232 (48.4%) received isolated mAFP support, 59 (12.3%) were treated with isolated VA-ECMO, and 64 (13.4%) underwent ECMELLA support (Fig. 1). Overall, in-hospital mortality was 42.6% (204/479). Among the 275 patients surviving the index hospitalization, 188 (68.4%) were transferred to a rehabilitation facility. Complete data for all six final model predictors were available for 416 patients (86.8%); variable-specific availability and missingness are reported in Supplementary Table 3. Patients with incomplete predictor data had higher in-hospital mortality than complete cases (84.1% [53/63] vs 36.3% [151/416]; p < 0.001). A similar pattern was observed in the development cohort, with mortality rates of 84.4% (38/45) and 35.8% (105/293), respectively.

**Fig. 1:**
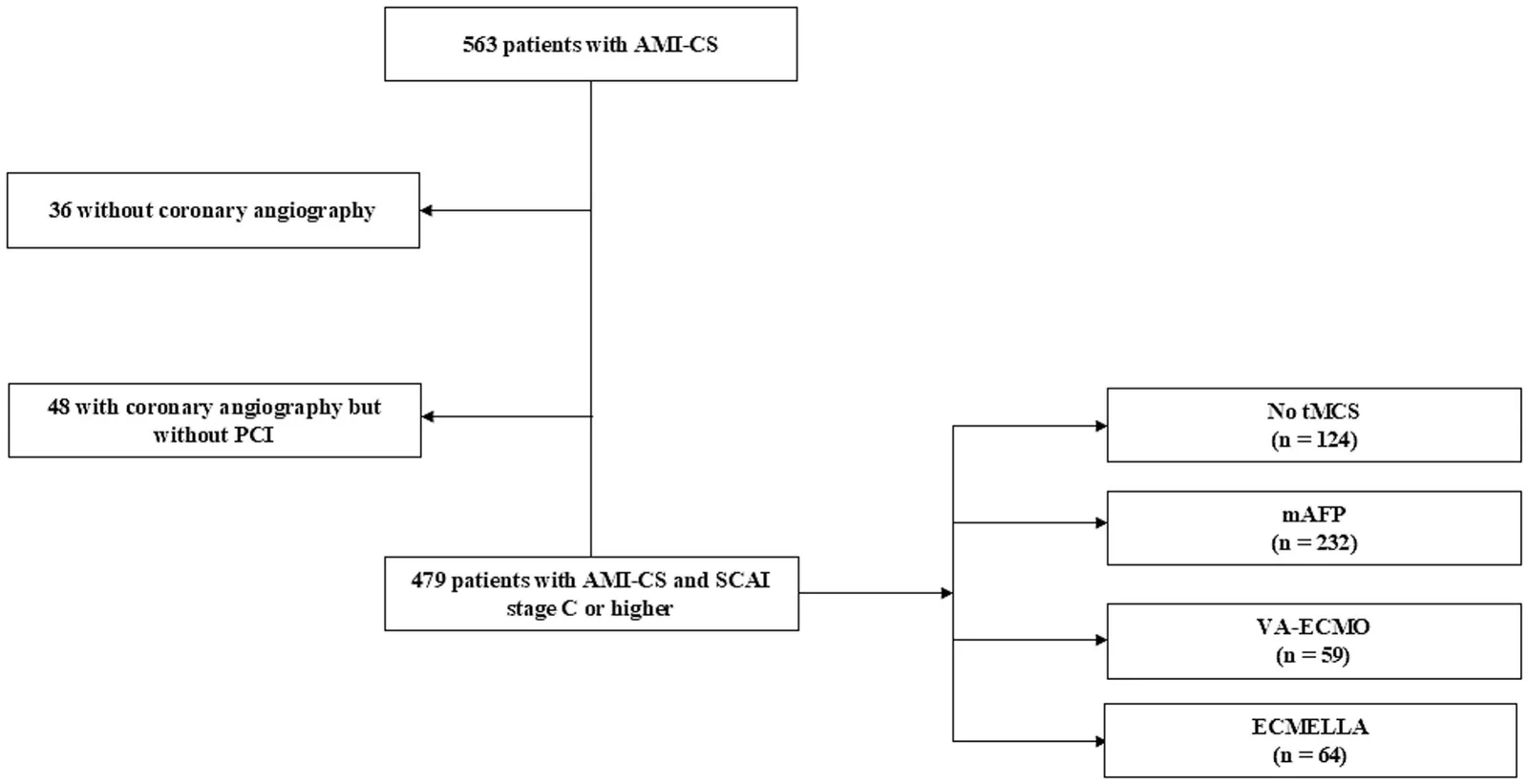
Study population flowchart.

Baseline characteristics differed substantially between survivors and non-survivors (Table 1). Non-survivors were older, had more comorbidities, and more frequently presented with NSTEMI, left main culprit lesions, incomplete post-PCI TIMI 3 flow, and greater shock severity, reflected by higher lactate and VIS, lower eGFR and hemoglobin, and more frequent invasive mechanical ventilation, CRRT, VA-ECMO, and ECMELLA. Among patients undergoing CPR with available duration data, median CPR duration was shorter in survivors than in non-survivors. GRACE, IABP-SHOCK II, and CardShock scores were higher among non-survivors, whereas ENCOURAGE did not differ significantly.

**Table 1:** Baseline characteristics according to in-hospital survival status. Percentages are based on available data for each variable.

Variables	Survivors (n = 275)	Non-survivors (n = 204)	p–value
**Demographics and comorbidities**			
Age (years)^2^	66.7 (±12.0)	73.2 (±10.9)	<0.001
Male sex^1^	203 (73.8)	138 (67.6)	0.140
BMI (kg/m^2^)^2^	28.2 (±4.6)	27.9 (±5.0)	0.485
Charlson comorbidity index^2^	3.5 (±2.3)	5.1 (±2.3)	<0.001
Known coronary artery disease^1^	57 (20.7)	69 (33.8)	0.001
Prior myocardial infarction^1^	43 (15.6)	51 (25.0)	0.011
Prior PCI^1^	49 (17.8)	49 (24.0)	0.096
Prior CABG^1^	16 (5.8)	17 (8.3)	0.283
Atrial fibrillation^1^	29 (10.5)	36 (17.6)	0.025
Arterial hypertension^1^	161 (58.5)	145 (71.1)	0.005
Diabetes mellitus^1^	71 (25.8)	71 (34.8)	0.033
Hyperlipidemia^1^	63 (22.9)	54 (26.5)	0.370
Smoking history (>5 pack–years)^1^	97 (35.3)	55 (27.0)	0.053
Chronic kidney disease (KDIGO stage ≥3a)^1^	38 (13.8)	45 (22.1)	0.018
**Coronary angiography and infarct characteristics**			
STEMI^1^	180 (65.5)	106 (52.0)	0.003
NSTEMI^1^	95 (34.5)	98 (48.0)	0.003
Single–vessel coronary artery disease^1^	64 (23.3)	34 (16.7)	0.076
Multivessel coronary artery disease^1^	211 (76.7)	170 (83.3)	0.076
PCI extent^1^			0.041
Single–vessel PCI	211 (76.7)	138 (68.3)	
Multivessel PCI	64 (23.3)	64 (31.7)	
Post–PCI TIMI 3 flow^1^	256 (96.2)	173 (87.8)	<0.001
Culprit vessel: left main coronary artery^1^	23 (8.5)	36 (17.6)	0.003
Culprit vessel: left anterior descending artery^1^	176 (64.7)	124 (60.8)	0.380
Culprit vessel: left circumflex artery^1^	65 (23.9)	67 (32.8)	0.031
Culprit vessel: right coronary artery^1^	80 (29.4)	56 (27.5)	0.639
Culprit vessel: bypass graft^1^	5 (1.8)	9 (4.4)	0.100
**Resuscitation characteristics**			
CPR^1^	116 (42.2)	139 (68.1)	<0.001
Out–of–hospital CPR^1^	77 (28.0)	65 (31.9)	0.360
Shockable rhythm at first documented rhythm assessment (VF/pVT)^1^	85 (31.4)	53 (26.8)	0.281
Witnessed collapse^1^	99 (36.3)	125 (61.6)	<0.001
CPR duration among patients undergoing CPR (min)^3^	15.0 [6.0–30.0]	23.0 [9.0–45.0]	0.032
**Mechanical circulatory support**			
Isolated VA–ECMO support^1^	23 (8.4)	36 (17.6)	<0.001
Isolated mAFP support^1^	153 (55.6)	79 (38.7)	<0.001
ECMELLA support^1^	22 (8.0)	42 (20.6)	<0.001
**Organ support therapies**			
Invasive mechanical ventilation^1^	160 (58.2)	172 (84.3)	<0.001
Non–invasive ventilation^1^	66 (24.0)	44 (21.6)	0.532
CRRT^1^	83 (30.2)	107 (52.5)	<0.001
Duration of CRRT (h)^3^	0.0 [0.0–74.0]	4.0 [0.0–72.0]	0.004
Duration of mechanical ventilation (h)^3^	117.0 [0.0–357.8]	37.0 [6.0–170.0]	0.136
Hospital length of stay (days)^2^	17.9 (±10.5)	7.4 (±8.6)	<0.001
**ICU parameters at admission**			
Left ventricular ejection fraction (%)^2^	40.4 (±11.4)	37.7 (±11.5)	0.019
Lactate (mmol/L)^3^	2.6 [1.4–5.5]	6.0 [2.4–11.0]	<0.001
pH^3^	7.36 [7.27–7.42]	7.30 [7.14–7.39]	<0.001
Base excess (mmol/L)^3^	−4.3 [−8.0 to −2.0]	−7.5 [−14.1 to −3.5]	<0.001
Creatinine (mg/dL)^3^	1.2 [0.9–1.5]	1.4 [1.1–2.0]	<0.001
Estimated glomerular filtration rate (mL/min/1.73 m^2^)^3^	57.0 [43.0–75.0]	45.5 [28.0–61.0]	<0.001
Hemoglobin (g/L)^3^	140.0 [124.0–152.0]	121.0 [101.0–139.0]	<0.001
Leukocyte count (/nL)^3^	13.6 [10.3–18.0]	13.8 [10.0–18.3]	0.856
Platelet count (/nL)^3^	240.0 [196.0–299.0]	212.5 [158.0–267.0]	<0.001
C–reactive protein (mg/dL)^3^	5.6 [2.4–20.3]	12.2 [3.1–50.6]	0.005
Procalcitonin (ng/mL)^3^	0.1 [0.1–0.2]	0.1 [0.1–0.3]	0.041
VIS^3^	8.9 [3.6–24.2]	26.6 [3.6–105.4]	<0.001
**Risk and severity scores at admission**			
SOFA score^2^	6.7 (±3.9)	8.1 (±4.5)	0.001
Glasgow Coma Scale score^2^	8.8 (±6.0)	7.8 (±5.9)	0.078
Horovitz index (mmHg)^3^	285.3 [161.4–477.1]	255.0 [130.5–421.9]	0.079
Shock index^3^	0.7 [0.5–0.9]	0.7 [0.6–1.0]	0.057
GRACE score^2^	172.0 (±26.0)	188.8 (±22.1)	<0.001
IABP-SHOCK II score^2^	1.8 (±1.6)	3.1 (±1.6)	<0.001
CardShock score^2^	3.0 (±1.5)	4.4 (±1.7)	<0.001
ENCOURAGE score, isolated VA-ECMO-treated patients only^2^	25.7 (±7.5)	27.2 (±7.5)	0.331

**Abbreviations:** BMI, body mass index; CABG, coronary artery bypass grafting; CPR, cardiopulmonary resuscitation; CRRT, continuous renal replacement therapy; ECMELLA, combined VA-ECMO and mAFP support; GRACE, Global Registry of Acute Coronary Events; ICU, intensive care unit; IABP, intra-aortic balloon pump; IQR, interquartile range; KDIGO, Kidney Disease: Improving Global Outcomes; mAFP, microaxial flow pump; NSTEMI, non-ST-segment elevation myocardial infarction; PCI, percutaneous coronary intervention; pVT, pulseless ventricular tachycardia; SOFA, Sequential Organ Failure Assessment; STEMI, ST-segment elevation myocardial infarction; TIMI, Thrombolysis In Myocardial Infarction; VA-ECMO, veno-arterial extracorporeal membrane oxygenation; VF, ventricular fibrillation; VIS, vasoactive–inotropic score. The Horovitz index was calculated as the ratio of arterial partial pressure of oxygen to the fraction of inspired oxygen and is reported in mmHg. The shock index was calculated as heart rate divided by systolic arterial pressure. The VIS was calculated as dopamine dose (μg/kg/min) + dobutamine dose (μg/kg/min) + 100 × epinephrine dose (μg/kg/min) + 10 × milrinone dose (μg/kg/min) + 100 × norepinephrine dose (μg/kg/min) + 10,000 × vasopressin dose (units/kg/min). eGFR was calculated using the 2021 race-free CKD-EPI creatinine equation. PCI extent was available in 477 patients (275 survivors and 202 non-survivors); percentages are based on available data. CPR duration was available in 228 of 255 patients undergoing CPR (103 survivors and 125 non-survivors). Superscripts indicate data presentation and the statistical test used: ^1^n (%) and Pearson’s chi-square test; ^2^mean (standard deviation) and Student’s t-test; and ^3^median [interquartile range] and the Mann–Whitney U test.

Treatment strategy was associated with marked differences in in-hospital outcome. In-hospital mortality differed significantly across support strategies (p < 0.001), with mortality rates of 37.9% in patients managed without tMCS, 34.1% in those receiving mAFP support, 61.0% in patients treated with isolated VA-ECMO, and 65.6% in those undergoing ECMELLA support (Fig. 2).

**Fig. 2:**
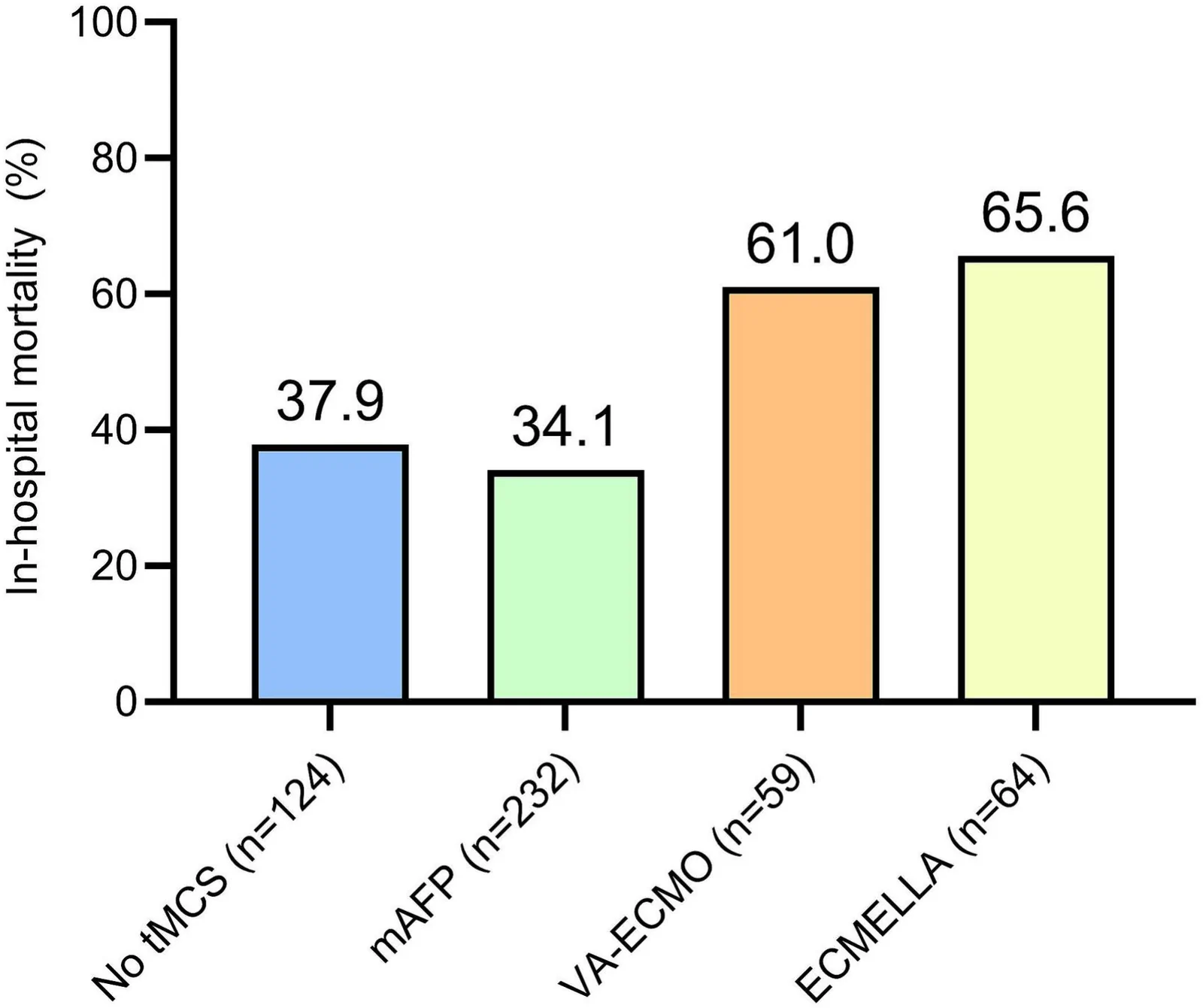
In-hospital mortality according to temporary mechanical circulatory support strategy.

### 3.2 Development and temporal validation of the admission-based prediction model

The development and temporal validation cohorts comprised 338 and 141 patients, respectively. The cohorts were broadly comparable regarding age, sex, prior CPR, LVEF, eGFR, VIS, complete-data availability, and in-hospital mortality (Supplementary Table 4). The temporal validation cohort included a higher proportion of patients presenting with STEMI (67.4% vs 56.5%; p = 0.027) and showed a different distribution of support strategies (overall p = 0.037), including fewer patients managed without tMCS. The proportions of complete cases (87.2% vs 86.7%, p = 0.872) and in-hospital mortality (43.3% vs 42.3%, p = 0.847) were similar between cohorts.

Following multiple imputation, model development was performed in all 338 patients from the 2017–2022 development cohort, including 143 in-hospital deaths. The pooled continuous AMICS-ADMIT model included age (OR 1.083 per year; 95% CI 1.053–1.113; p < 0.001), LVEF (OR 0.969 per 1% increase; 95% CI 0.946–0.993; p = 0.013), eGFR (OR 0.989 per mL/min/1.73 m^2^ increase; 95% CI 0.978–0.999; p = 0.039), VIS (OR 1.010 per point; 95% CI 1.005–1.016; p < 0.001), prior CPR (OR 3.960; 95% CI 2.182–7.186; p < 0.001), and STEMI status (OR 0.604; 95% CI 0.361–1.009; p = 0.054). The complete pooled coefficients and intercept are provided in Supplementary Table 2. Box–Tidwell transformation terms provided no evidence of departure from linearity in the log-odds for age (p = 0.674), LVEF (p = 0.692), eGFR (p = 0.570), or VIS (p = 0.331). The model showed good discrimination in the development cohort, with an AUC of 0.809 (95% CI 0.764–0.855) and a Brier score of 0.176. The calibration slope was 1.014, calibration-in-the-large was 0.001 (95% CI −0.252 to 0.254), and the jointly estimated calibration intercept was 0.004. Bootstrap internal validation demonstrated limited optimism, with a mean AUC optimism of 0.014 and an optimism-corrected AUC of 0.795. The optimism-corrected Brier score was 0.184 and the optimism-corrected calibration slope was 0.941. Predictor coefficients were directionally stable across bootstrap samples; notably, the STEMI coefficient remained negative in 97.3% of bootstrap fits**.** Application of the fixed pooled coefficients to the temporal validation cohort demonstrated good discrimination, with an AUC of 0.834 (95% CI 0.769–0.899) and a Brier score of 0.167. Overall calibration metrics were favourable, with a calibration slope of 1.075, calibration-in-the-large of 0.008 (95% CI −0.391 to 0.408), and a jointly estimated calibration intercept of 0.028. However, visual calibration showed local deviation in the temporal validation cohort, particularly in the third risk quintile, where observed mortality exceeded predicted mortality (Supplementary Fig. 1). In the sensitivity analysis excluding LVEF, the five-predictor model showed an AUC of 0.798 (95% CI 0.751–0.845) in the development cohort and 0.828 (95% CI 0.762–0.894) in temporal validation. The corresponding Brier scores were 0.181 and 0.167, respectively. In a complete-case sensitivity analysis restricted to the 293 development-cohort patients with complete data for all six predictors (105 deaths), the six-predictor model refitted in complete cases showed similar coefficient directions and magnitudes to the multiply imputed primary model. Discrimination remained comparable (AUC 0.812, 95% CI 0.763–0.861), with a Brier score of 0.169.

### 3.3 Derivation and performance of the AMICS-ADMIT bedside score

To facilitate clinical application, the final AMICS-ADMIT prediction model was converted into a simplified bedside score based on six categorized admission variables: age, LVEF, eGFR, VIS, prior CPR, and STEMI status (Table 2). Point allocation followed the coefficient-informed clinical simplification described in the Methods; no single universal scaling constant was applied. Across the 50 imputed development datasets, the pooled score coefficient was 0.47386, corresponding to an OR of 1.606 per point (95% CI 1.419–1.818; p < 0.001), and the pooled intercept was −3.16665. In the full multiply imputed development cohort (n = 338), the bedside score demonstrated an AUC of 0.777 (95% CI 0.728–0.826) and a Brier score of 0.190. The calibration slope was 1.017, calibration-in-the-large was 0.000 (95% CI −0.244 to 0.244), and the jointly estimated calibration intercept was 0.004. Application of the fixed development score-to-risk equation to the full temporal validation cohort (n = 141) resulted in an AUC of 0.775 (95% CI 0.699–0.851) and a Brier score of 0.190. The calibration slope was 1.021, calibration-in-the-large was 0.016 (95% CI −0.363 to 0.395), and the jointly estimated calibration intercept was 0.020.

**Table 2:** Composition of the AMICS-ADMIT bedside score.

Variable	Category	Points
Age	<60	0
	60–69	1
	70–79	2
	≥80	3
LVEF (%)	≥40	0
	30–39	1
	20–29	2
	<20	3
eGFR (mL/min/1.73 m^2^)	≥60	0
	30–59	1
	<30	2
VIS	<10	0
	10–30	1
	>30	2
Prior CPR	No	0
	Yes	3
STEMI on ECG	Yes	0
	No	1

**Note:** Points were assigned through a coefficient-informed clinical simplification rather than by applying a universal coefficient-dividing constant or rounding formula. For continuous predictors, one point was added for each worsening category relative to the lowest-risk category. Prior CPR and absence of STEMI were assigned 3 and 1 points, respectively, based on the relative magnitude and direction of their pooled coefficients. All categories and point weights were defined in the development cohort and applied unchanged in temporal validation.

### 3.4 Risk stratification according to AMICS-ADMIT categories

In the multiply imputed development cohort, pooled estimates of in-hospital mortality were 13.8%, 44.3%, and 71.5% in the low-, intermediate-, and high-risk groups, respectively. When the predefined score and risk-group cut-offs were applied unchanged to the multiply imputed temporal validation cohort, the corresponding mortality estimates were 11.6%, 48.8%, and 69.5%, respectively (Fig. 3). Compared with the low-risk group, mortality was significantly higher in both the intermediate- and high-risk groups in the development and temporal validation cohorts (all p < 0.001).

**Fig. 3:**
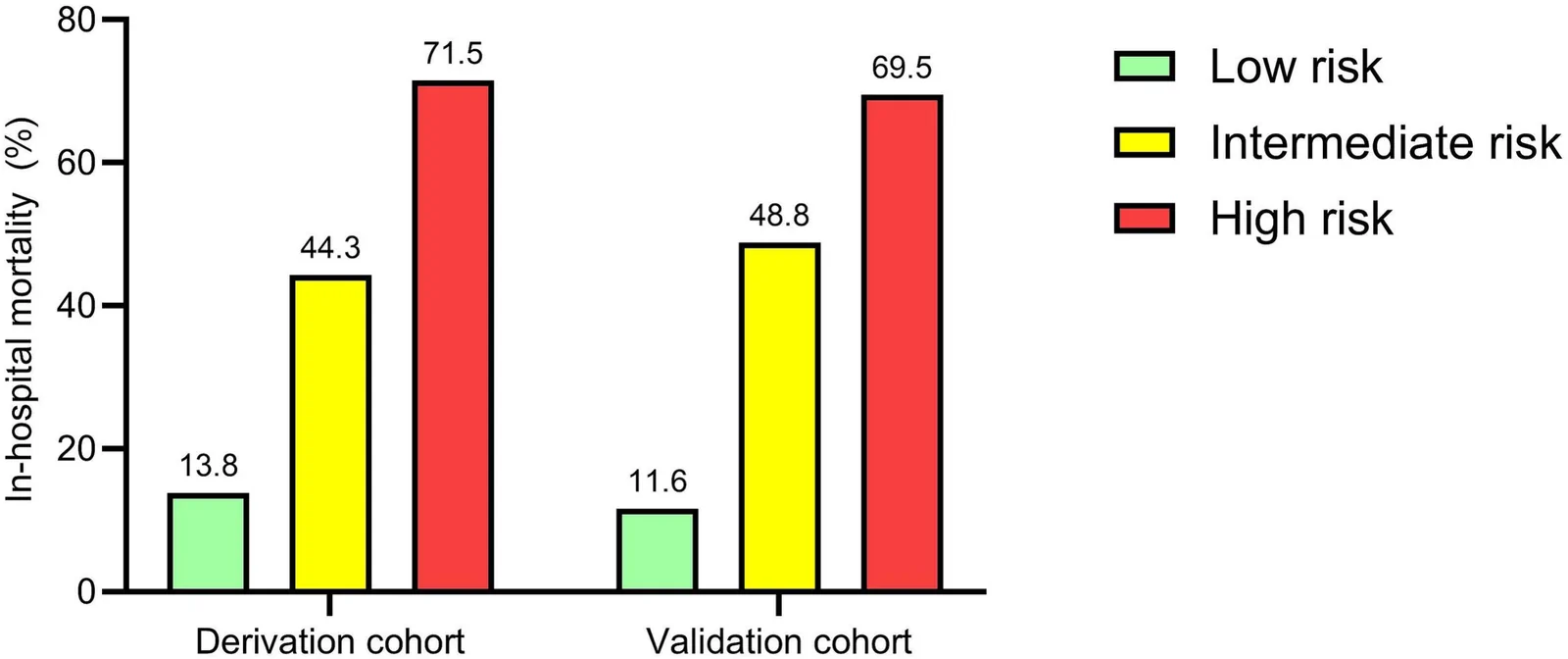
Pooled estimates of in-hospital mortality across AMICS-ADMIT risk categories in the multiply imputed development and temporal validation cohorts.

### 3.5 Benchmarking against established scores

Benchmarking of the AMICS-ADMIT bedside score against established scores was performed exclusively in the 2023–2025 temporal validation cohort. Pairwise analyses were restricted to patients with available data for the respective comparator. AMICS-ADMIT and CardShock showed similar discrimination (n = 111; 41 deaths; AUC 0.779 [95% CI 0.693–0.865] vs. 0.766 [0.667–0.866]; ΔAUC = 0.013; p = 0.706). Similarly, no significant difference was observed between AMICS-ADMIT and IABP-SHOCK II (n = 119; 49 deaths; AUC 0.784 [0.703–0.865] vs. 0.805 [0.729–0.880]; ΔAUC = −0.021; p = 0.632) or GRACE (n = 111; 42 deaths; AUC 0.780 [0.694–0.866] vs. 0.723 [0.622–0.825]; ΔAUC = 0.056; p = 0.307). Accordingly, these analyses indicate broadly comparable discrimination in temporal validation and are interpreted as descriptive benchmarking rather than evidence of superiority.

### 3.6 Performance across tMCS strata

In exploratory analyses of the overall cohort, the AMICS-ADMIT score showed an AUC of 0.776 (95% CI 0.735–0.817). Across tMCS strata, discrimination was 0.757 (95% CI 0.669–0.844) in patients managed without tMCS and 0.811 (95% CI 0.755–0.867) in patients receiving mAFP support. Numerically lower discrimination was observed in patients treated with isolated VA-ECMO (AUC 0.664; 95% CI 0.529–0.799) and ECMELLA (AUC 0.656; 95% CI 0.515–0.797). In the isolated VA-ECMO subgroup, ENCOURAGE showed an AUC of 0.678 (95% CI 0.507–0.849). Established scores showed heterogeneous performance across treatment strata, with wide confidence intervals particularly in the smaller VA-ECMO and ECMELLA subgroups (Fig. 4). These treatment-stratified analyses were exploratory and were not intended as formal head-to-head comparisons between prediction models.

**Fig. 4:**
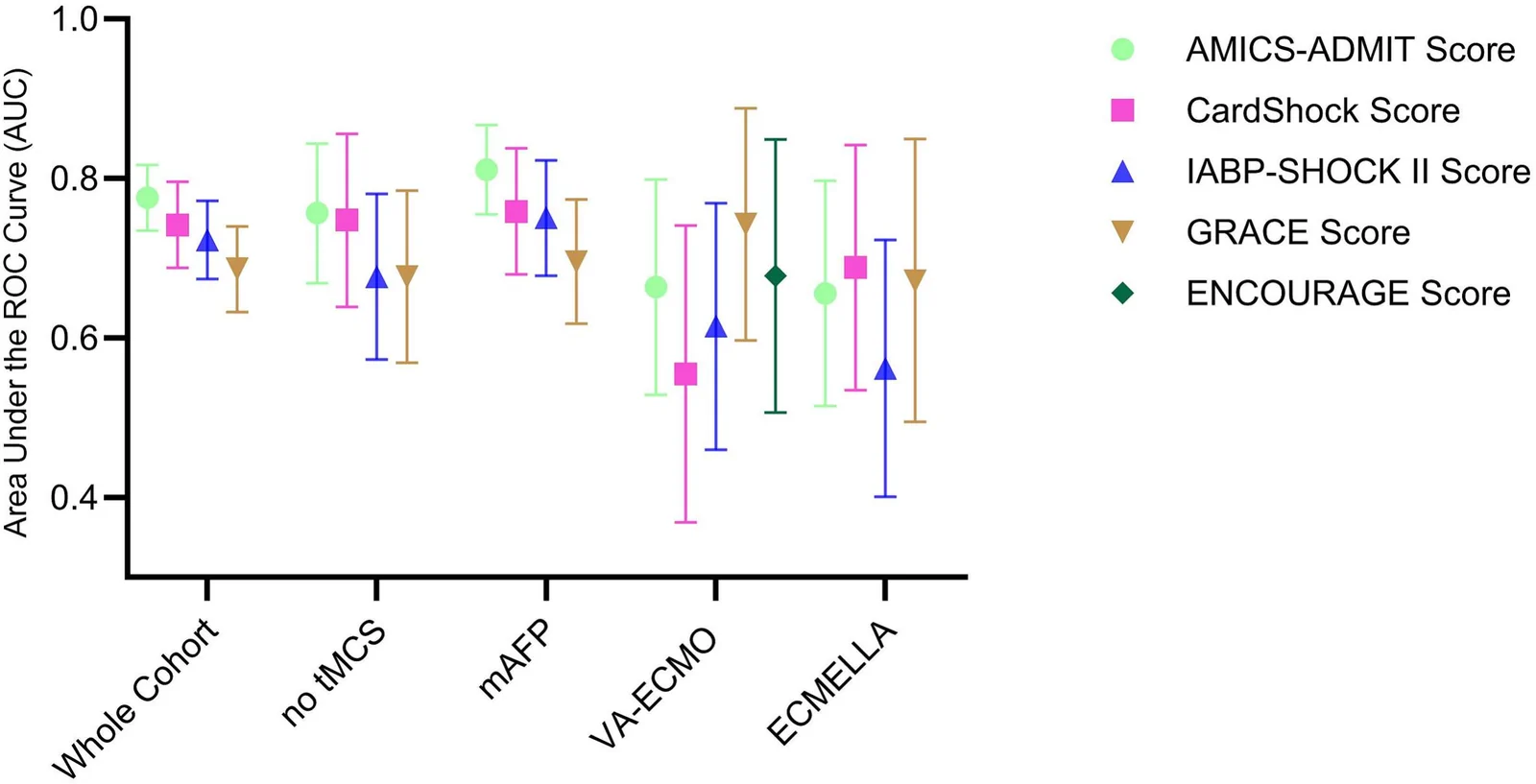
Exploratory discrimination of AMICS-ADMIT bedside score and established risk scores across temporary mechanical circulatory support strata.

## 4 Discussion

In this contemporary PCI-treated AMI-CS device-era cohort, we developed and temporally validated AMICS-ADMIT, an admission-based mortality prediction model translated into a simplified bedside score. Based on age, LVEF, prior CPR, eGFR, VIS, and STEMI status, the model showed good discrimination and temporal stability. The bedside score preserved clinically meaningful risk stratification, separating low-, intermediate-, and high-risk patients by observed mortality. Descriptive benchmarking suggested performance comparable to established scores, with preserved discrimination in no-tMCS and mAFP patients but attenuated performance in VA-ECMO and ECMELLA cohorts.

Early risk stratification remains a central unmet need in AMI-CS [20]. Despite advances in revascularization, intensive care, and device-based support, short-term mortality remains high and key therapeutic decisions are often required before the trajectory of multiorgan failure and treatment response becomes apparent [21], [22], [23], [24], [25], [26]. Existing scores have contributed substantially to prognostic assessment, but differ in derivation cohorts, treatment eras, included variables, and intended populations. CardShock was derived in broad CS, IABP-SHOCK II in infarct-related CS, GRACE in ACS, and ENCOURAGE in VA-ECMO-treated AMI-CS. AMICS-ADMIT was therefore designed as a pragmatic admission-based framework for early risk estimation across contemporary AMI-CS treatment pathways.

A central aspect of AMICS-ADMIT is that it was first derived as a continuous admission-based prediction model and subsequently translated into a simplified bedside score. Following multiple imputation in the full development cohort, the continuous model showed good discrimination in the development cohort (AUC 0.809) and temporal validation cohort (AUC 0.834), with overall favourable calibration metrics and Brier scores, although visual calibration showed local deviation in the temporal validation cohort. The simplified bedside score likewise demonstrated stable discrimination across the multiply imputed development and temporal validation cohorts, with AUCs of 0.777 and 0.775, respectively, Brier scores of 0.190 in both cohorts, and calibration slopes close to unity. As expected, the pragmatic simplification through categorization and integer weighting, rather than a strictly proportional translation of the regression coefficients, was associated with a modest reduction in discrimination compared with the continuous model (AUC 0.777 vs. 0.809), representing a trade-off between predictive information and bedside usability.

The six AMICS-ADMIT components reflect complementary dimensions of early AMI-CS severity: age captures baseline vulnerability, LVEF acute myocardial dysfunction, prior CPR pre-admission circulatory collapse, eGFR renal dysfunction and comorbidity burden, VIS pharmacological circulatory support intensity, and STEMI status differences in infarct phenotype and treatment pathways. The association of STEMI with lower mortality should not be interpreted causally, but likely reflects differences in comorbidity burden, time-to-recognition, coronary pathways, and treatment selection between STEMI- and NSTEMI-related shock. In addition, because inclusion required PCI, selection bias may have contributed to this association. Particularly unstable patients with STEMI who died before coronary angiography or before PCI could be performed would not have entered the study cohort, potentially selecting for STEMI patients who survived to revascularization. Because all variables are routinely documented during the early hospital course, AMICS-ADMIT can be calculated without specialized biomarkers or complex dynamic modelling. However, it should not be interpreted as a strictly pre-treatment model, because some predictor measurements may have followed initial resuscitation, PCI, vasoactive escalation, or tMCS initiation. Accordingly, AMICS-ADMIT is intended for early in-hospital risk stratification once these routinely collected variables are available, rather than for risk estimation before initial resuscitative or interventional treatment. The observed risk gradient supported the clinical interpretability of AMICS-ADMIT. In the multiply imputed development cohort, pooled mortality estimates increased from 13.8% in the low-risk group to 44.3% and 71.5% in the intermediate- and high-risk groups, respectively. The corresponding estimates in the temporal validation cohort were 11.6%, 48.8%, and 69.5%. This stratification may support early bedside communication, documentation of baseline risk, and harmonization of risk profiles across cohorts and future studies, while complementing rather than replacing repeated clinical reassessment.

The comparison with established scores should be interpreted as descriptive benchmarking rather than evidence that AMICS-ADMIT replaces existing tools. To avoid favouring the newly developed model, the principal comparisons with CardShock, IABP-SHOCK II, and GRACE were restricted to the independent 2023–2025 temporal validation cohort using identical paired samples. No significant differences in discrimination were observed between AMICS-ADMIT and any of these comparator scores. These findings should be interpreted in light of important differences in derivation populations and prediction horizons: CardShock was developed in a broad CS population for in-hospital mortality, IABP-SHOCK II in AMI-CS for 30-day mortality, and GRACE in ACS for 6-month post-discharge mortality [27]. Accordingly, the present analyses provide contextual benchmarking rather than evidence of superiority. In the isolated VA-ECMO subgroup, AMICS-ADMIT and ENCOURAGE showed similar discrimination; however, this comparison remains exploratory because of the small and highly selected subgroup.

The exploratory tMCS-stratified analyses clarify the potential scope of AMICS-ADMIT. Discrimination was preserved in patients managed without tMCS and in those treated with mAFP support, suggesting that admission variables retain substantial prognostic information in these groups. In contrast, performance was attenuated in patients receiving VA-ECMO or ECMELLA. However, the estimates in the isolated VA-ECMO and ECMELLA subgroups were based on small, highly selected populations and were accompanied by wide confidence intervals. The apparent attenuation may therefore reflect statistical imprecision, sampling variability, treatment-selection bias, and confounding by indication rather than a stable difference in model performance. Prognosis in these advanced-support populations may also depend more strongly on dynamic factors such as the timing of support, unloading, neurological injury, complications, inflammatory response, and early organ recovery [28]. These findings should therefore be considered exploratory and hypothesis-generating. Recent CS risk-prediction approaches, including machine-learning and dynamic models, may improve prediction by incorporating serial variables or larger data streams [29], [30]. However, AMICS-ADMIT was intentionally designed as a low-complexity bedside tool that can be calculated from routinely documented variables obtained during early hospital care.

Taken together, these findings position AMICS-ADMIT as a pragmatic admission-based model and bedside score for early risk stratification in contemporary PCI-treated AMI-CS. Its main strength is the combination of a continuous prediction model with a clinically interpretable simplified score. For individual clinical application, all six predictor values are required; AMICS-ADMIT was not designed to impute unavailable predictors at the bedside. The score is intended for use by healthcare professionals involved in the care of patients with AMI-CS and does not require specialized analytical software. Rather than replacing dynamic reassessment, shock-team evaluation, or device-specific decision-making, AMICS-ADMIT provides an estimate of mortality risk based on variables obtained during early hospital care and should be interpreted alongside subsequent treatment response and clinical evolution.

## 5 Limitations

This study has limitations inherent to its retrospective, single-centre design. Although the cohort reflects contemporary AMI-CS care in a tertiary cardiac intensive care centre with broad availability of tMCS, centre-specific referral pathways, treatment algorithms, device availability, and thresholds for escalation may limit generalizability. Moreover, temporal validation was performed within the same institution and therefore does not account for centre-specific practice patterns; external multicentre validation remains necessary before routine clinical implementation. In addition, patients who did not undergo coronary angiography and those who underwent coronary angiography without PCI were excluded. Accordingly, AMICS-ADMIT was developed and validated in a selected cohort of AMI-CS patients undergoing PCI, which may limit its generalizability to patients managed without invasive coronary evaluation or revascularization. In addition, SCAI shock severity was assessed retrospectively, and formal subclassification into stages C, D, and E could not be reconstructed reliably for all patients at a uniform index time. The use of bivariable screening and backward elimination may introduce model-selection instability. In addition, the available development cohort of 338 patients with 143 deaths was modestly smaller than the sample size suggested by the post hoc Riley-based assessment, which may increase susceptibility to model optimism and coefficient instability. Furthermore, categorization of continuous predictors and pragmatic integer weighting inevitably result in some loss of information and discrimination relative to the continuous model; the bedside score should therefore be regarded as a clinically oriented simplification requiring external multicentre validation. Although multiple imputation enabled cohort-wide evaluation of bedside-score discrimination, calibration, and risk-group separation, direct calculation of the score in routine clinical practice requires all six component variables to be available. Treatment allocation was not randomized, and the use and type of tMCS were determined by the treating physicians. Device-stratified analyses are therefore susceptible to confounding by indication, including shock severity, neurological status, comorbidity burden, expected reversibility, and perceived procedural benefit. These analyses should be interpreted as exploratory assessments of score performance across contemporary support pathways rather than evidence of treatment effects or guidance for selecting a specific support strategy. Missing predictor data, particularly for LVEF, were handled using multiple imputation before model estimation in the development cohort, thereby avoiding restriction of the primary analysis to complete cases. Nevertheless, the missing-at-random assumption cannot be verified, and residual bias related to missing or retrospectively collected admission variables cannot be excluded. Owing to the retrospective study design, the exact timing of LVEF, eGFR, and VIS assessment relative to return of spontaneous circulation, PCI, vasoactive escalation, and tMCS implantation could not be reliably reconstructed at the individual-patient level. Some LVEF measurements may have been obtained during or after mAFP or VA-ECMO implantation. The predictors should therefore be interpreted as early admission variables rather than strictly pre-treatment measurements. Model fairness across broader sociodemographic groups was not formally assessed and should be examined in future external multicentre validation studies. The primary endpoint was all-cause in-hospital mortality, which is clinically relevant and reliably ascertainable but may be influenced by length of stay, transfer practices, and institutional end-of-life decision-making. Systematic follow-up beyond discharge or transfer was not available; therefore, the performance of AMICS-ADMIT for longer-term outcomes could not be assessed. Finally, AMICS-ADMIT was intentionally designed as an admission-based tool and does not include dynamic markers of treatment response, evolving organ failure, neurological recovery, or device-related complications. It should therefore support early baseline risk estimation and cohort characterization, while complementing rather than replacing repeated clinical reassessment, shock-team judgement, and device-specific decision-making.

## 6 Conclusions

AMICS-ADMIT provides a temporally validated admission-based model and simplified bedside score for early in-hospital mortality risk stratification in contemporary PCI-treated AMI-CS. Using six routinely documented variables obtained during early hospital care, it provides an admission-based estimate of mortality risk while complementing subsequent assessment of treatment response and evolving organ failure. Pending external multicentre validation, AMICS-ADMIT may support early risk assessment, shock-team communication, cohort characterization, and future risk-guided research while complementing dynamic clinical reassessment.

## CRediT authorship contribution statement

**Klevis Mihali:** Writing – review & editing, Writing – original draft, Visualization, Validation, Software, Resources, Methodology, Investigation, Formal analysis, Data curation, Conceptualization. **Birgit Markus:** Writing – review & editing, Writing – original draft, Supervision, Conceptualization. **Kornelia Neuner:** Writing – review & editing, Data curation. **Georgios Chatzis:** Writing – review & editing. **Lukas Harbaum:** Writing – review & editing. **Mykyta Fomin:** Writing – review & editing. **Maryana Choukeir:** Writing – review & editing. **Styliani Syntila:** Writing – review & editing. **Bernhard Schieffer:** Writing – review & editing, Writing – original draft, Supervision, Project administration, Conceptualization. **Julian Kreutz:** Writing – review & editing, Writing – original draft, Visualization, Validation, Supervision, Software, Project administration, Methodology, Investigation, Formal analysis, Data curation, Conceptualization.

## Informed consent statement

Patient consent was waived due to the retrospective, non-interventional design of the study.

## Institutional review board statement

The study was approved by the Ethics Committee of the Faculty of Medicine, Philipps University Marburg (No. 26-284 RS; 14 August 2026) and was conducted in accordance with the Declaration of Helsinki. Owing to the retrospective, non-interventional design, the requirement for individual informed consent was waived.

## Funding

Open Access funding provided by the Open Access Publishing Fund of Philipps-Universität Marburg.

## Declaration of competing interest

The authors declare the following financial interests/personal relationships which may be considered as potential competing interests: Julian Kreutz, Georgios Chatzis, Bernhard Schieffer and Birgit Markus received speakers’ honoraria from Abiomed; and Birgit Markus received research funding from Abiomed. The remaining authors declare no competing interests.

## Data Availability

The data presented in this study are available from the corresponding author upon reasonable request. The data are not publicly available due to patient privacy and data protection restrictions.
