# Supplementary material for: Mortality risk stratification in contemporary myocardial infarction–related cardiogenic shock: development of AMICS-ADMIT prediction model and benchmarking of established scores
Source: Int J Cardiol Heart Vasc. 2026 Oct 14;67:102029. doi: 10.1016/j.ijcha.2026.102029 (PMC13683238; doi:10.1016/j.ijcha.2026.102029)
Supplement: Supplementary file 1 [file mmc1.docx]

Supplement

**Supplementary Figure 1.** Calibration of the primary multiply imputed continuous AMICS-ADMIT model in the development cohort (left) and temporal validation cohort (right). Points represent observed in-hospital mortality across quintiles of patient-level mean predicted probability, with vertical error bars indicating 95% Wilson confidence intervals. The diagonal dotted line indicates perfect calibration.

**Supplementary Table 1.** Unadjusted associations between candidate predictors and in-hospital mortality in the 2017–2022 development cohort. Odds ratios, 95% confidence intervals, and p-values are reported for each candidate predictor.

| **Candidate predictor** | **Unadjusted OR (95% CI)** | **p-value** |
| --- | --- | --- |
| Age, per year | 1.048 (1.027–1.069) | <0.001 |
| Male sex | 0.727 (0.452–1.170) | 0.189 |
| Known coronary artery disease | 2.022 (1.258–3.251) | 0.004 |
| Chronic heart failure | 2.145 (1.022–4.503) | 0.044 |
| eGFR, per 1 mL/min/1.73 m² | 0.977 (0.967–0.987) | <0.001 |
| LVEF, per 1% increase | 0.976 (0.956–0.997) | 0.027 |
| STEMI | 0.505 (0.325–0.783) | 0.002 |
| Prior CPR | 2.536 (1.619–3.972) | <0.001 |
| CPR duration, per minute | 1.028 (1.016–1.041) | <0.001 |
| Initial PEA/asystole | 5.909 (3.384–10.317) | <0.001 |
| Lactate, per 1 mmol/L | 1.119 (1.067–1.173) | <0.001 |
| MAP, per 1 mmHg | 0.985 (0.975–0.995) | 0.004 |
| VIS, per point | 1.009 (1.005–1.014) | <0.001 |

**Supplementary Table 2.** Pooled coefficients of the primary six-predictor AMICS-ADMIT model derived after multiple imputation in the 2017–2022 development cohort. The model intercept is provided to enable calculation of individual predicted probabilities.

| **Predictor** | **β** | **Standard error** | **Adjusted OR (95% CI)** | **p-value** |
| --- | --- | --- | --- | --- |
| Intercept | −4.93202 | 1.22187 | — | <0.001 |
| Age, per year | 0.07946 | 0.01398 | 1.083 (1.053–1.113) | <0.001 |
| LVEF, per 1% increase | −0.03127 | 0.01259 | 0.969 (0.946–0.993) | 0.013 |
| eGFR, per mL/min/1.73 m² increase | −0.01153 | 0.00558 | 0.989 (0.978–0.999) | 0.039 |
| VIS, per point increase | 0.01041 | 0.00261 | 1.010 (1.005–1.016) | <0.001 |
| Prior CPR, yes versus no | 1.37630 | 0.30400 | 3.960 (2.182–7.186) | <0.001 |
| STEMI, yes versus no | −0.50489 | 0.26204 | 0.604 (0.361–1.009) | 0.054 |

**Supplementary Table 3*.*** Completeness of final model predictors and the outcome variable. Percentages are based on the total study population of 479 patients. Complete cases were defined as patients with available data for all six final model predictors and in-hospital mortality.

| **Variable** | **Available, n (%)** | **Missing, n (%)** |
| --- | --- | --- |
| Age | 479 (100.0) | 0 (0.0) |
| LVEF | 425 (88.7) | 54 (11.3) |
| eGFR | 465 (97.1) | 14 (2.9) |
| VIS | 479 (100.0) | 0 (0.0) |
| Prior CPR | 479 (100.0) | 0 (0.0) |
| STEMI status | 479 (100.0) | 0 (0.0) |
| In-hospital mortality | 479 (100.0) | 0 (0.0) |
| Complete data for all final predictors and outcome | 416 (86.8) | 63 (13.2) |

**Supplementary Table 4*.*** Comparison of the development and temporal validation cohorts. Data are presented as mean ± standard deviation, median [interquartile range], or n (%), as appropriate. P-values were calculated using Student’s t-test for age and LVEF, the Mann–Whitney U test for eGFR and VIS, and Pearson’s chi-square test for categorical variables. The p-value for support strategy represents the overall comparison across the four categories.

| **Variable** | **Development cohort**  **2017–2022** | **Temporal validation cohort 2023–2025** | **p–value** |
| --- | --- | --- | --- |
| Number of patients | 338 | 141 | — |
| Age, years | 69.3 ± 12.2 | 69.8 ± 11.2 | 0.715 |
| Male sex | 242 (71.6%) | 99 (70.2%) | 0.760 |
| STEMI | 191 (56.5%) | 95 (67.4%) | 0.027 |
| Prior CPR | 183 (54.1%) | 72 (51.1%) | 0.538 |
| LVEF, % | 40.0 ± 11.7 | 37.8 ± 10.7 | 0.059 |
| eGFR, mL/min/1.73 m² | 53.5 [37.8–70.0] | 52.0 [39.0–69.0] | 0.749 |
| VIS | 11.7 [3.3–38.2] | 19.2 [3.7–59.4] | 0.146 |
| **Support strategy** |  |  | 0.037 |
| No tMCS | 99 (29.3%) | 25 (17.7%) | — |
| mAFP | 159 (47.0%) | 73 (51.8%) | — |
| Isolated VA-ECMO | 41 (12.1%) | 18 (12.8%) | — |
| ECMELLA | 39 (11.5%) | 25 (17.7%) | — |
| Complete cases | 293 (86.7%) | 123 (87.2%) | 0.872 |
| In-hospital mortality | 143 (42.3%) | 61 (43.3%) | 0.847 |
